# 3D-printed resin composite posterior fixed dental prosthesis: a prospective clinical trial up to 1 year

**DOI:** 10.3389/fdmed.2024.1390600

**Published:** 2024-06-04

**Authors:** Paniz Hobbi, Tugce Merve Ordueri, Funda Öztürk-Bozkurt, Tugba Toz-Akalın, Muzaffer Ateş, Mutlu Özcan

**Affiliations:** ^1^ Department of Prosthetic Dentistry, School of Dentistry, Istanbul Medipol University, Istanbul, Türkiye; ^2^ Department of Restorative Dentistry, School of Dentistry, Istanbul Medipol University, Istanbul, Türkiye; ^3^ Department of Restorative Dentistry, School of Dentistry, Istinye University, Istanbul, Türkiye; ^4^ Clinic for Masticatory Disorders and Dental Biomaterials, Center for Dental Medicine, University of Zurich, Zurich, Switzerland

**Keywords:** clinical trial, dental materials, composite 3-unit posterior FDPs, prosthodontics, restorative materials, survival, 3D-printed restorations, permanent restorations

## Abstract

**Objectives:**

This clinical trial evaluated the clinical behavior of 3D-printed posterior resin composite fixed dental prostheses (FDPs).

**Materials and methods:**

Between 10 October 2020 and 5 August 2022, 49 patients aged 19–60 years (16 men, 33 women) received 68 3D-printed resin composite 3-unit posterior FDPs (ELS Even Stronger, Saremco, Switzerland). FDPs were followed up 2 weeks after placement (baseline), 6 months after placement, and 1 year after placement by two independent calibrated observers using modified FDI criteria for anatomical form, secondary caries, marginal adaptation, surface roughness, color match, fracture of material, staining surface, staining margin, approximate anatomical form, retention, gingival health, and patient's view. Survival analyses were performed using Kaplan–Meier analyses.

**Results:**

A total of 59 restorations were evaluated and the mean observation period was 8.63 months. Failure types were categorized as mechanical and biological. Failures were observed in 14 FDPs. Nine FDPs showed mechanical failure and five FDPs showed biological failure. Mechanical failures were mostly experienced as connector fractures. Eight FDPs showed cohesive fractures (seven in a single connector at either at the mesial or distal and one in the pontic itself). Biological failures, including root canal treatment and gingival tissue reactions, were observed within the first 6 months. Based on mechanical failures, the survival rate was 86.7% including the biological complications; this corresponded to 71.6% (Kaplan–Meier). After 1 year, two FDPs showed surface luster loss (score 4), two-color mismatches (score 4), and two-surface staining (score 4).

**Conclusion:**

3D-printed resin composite FDPs were observed acceptable after 1 year of clinical follow-up, providing that the experienced failure types were mainly associated with fractures in the connector region, which requires revision of design parameters.

**Clinical Trial Registration:**

Clinical Trials.Gov, NCT04600297.

## Introduction

Missing teeth not only cause functional and structural problems in patients, but also have a high impact on these individuals’ social and psychological states. This situation paved the way for the introduction of different treatment methods and/or the development of existing ones to eliminate the losses observed in oral environments. Computer-aided design/computer-aided manufacturing (CAD/CAM) is one of the steps of these current targets in dentistry (1). After the introduction of the CAD/CAM system to dental clinicians, the usage of resin composites as an alternative restorative material in contemporary, minimally invasive prosthodontics has increased dramatically. CAD/CAM systems offer both subtractive and additive manufacturing (AM). Indirect composite resin restorations were manufactured with a CAD/CAM workflow using subtractive manufacturing (SM), such as milling (2). However, milling or grinding allows fast and effective fabrication, but is also associated with high material loss and instrument wear. In addition, the milling process is time-consuming, and its accuracy depends on the geometry of the bur. These disadvantages of the subtractive technique have recently increased the use of three-dimensional (3D) printing in dentistry (3). 3D printing is emerging as a new technology that overcomes the limitations of subtractive manufacturing systems in dentistry with current developments in 3D printing materials and 3D printers (4). AM is a type of 3D manufacturing technology that builds materials by layering, thus producing virtually no waste material. There are also no restrictions on the geometric shapes of the products, and the tolerance of the milled parts is no longer an issue (5). This technique builds the object layer by layer with fewer restrictions for three-dimensional geometric shaping and 3D printable resin composite materials are typically built up layer by layer using digital light processing (DLP) technology (6). The integration of 3D printing into modern dentistry enables the fabrication of prosthodontic, orthodontic, and surgical devices requiring flexible and abrasion-resistant materials. Various additive manufacturing technologies utilize a range of materials, such as polymers, composites, ceramics, and metal alloys (7).

The advantages of resin composites, which are frequently preferred in digital dentistry, compared to glass ceramics, are their low abrasiveness on antagonist teeth (8), their ability to better absorb functional stresses (9), and their intraoral repairability (10). Clinical studies confirm the promising performance of such indirect resin composite restorations and demonstrate the potential of resin composite as a material that can address biomimetic principles of tissue preservation (11, 12).

The mechanical properties, such as fracture and fatigue strengths, of resin composite CAD/CAM materials have been evaluated in several *in vitro* studies (13, 14). In addition to this *in vitro* literature, the results of studies considering clinical performances of single crown restorations fabricated with resin-based CAD/CAM materials indicate that these treatment alternatives supported with teeth have a favorable short-term survival rate (15, 16). 3D printing technology contributes to the way dental restorations are created, allowing more precise and accurate restorations to be printed rather than traditional methods of creating dental restorations, such as using molds and physical models. This alternative treatment method can lead to better fitting and longer-lasting restorations. Enabling the prosthetic applicability of current resin composites, CAD/CAM and 3D printing are innovative and rapidly evolving technologies gaining popularity in the dental industry, providing greater accuracy, repeatability, speed, and cost-effectiveness. It has been stated that with the developments in materials and technologies used in dentistry, resin composites with more advanced properties will increase the long-term clinical stability of digitally manufactured indirect resin composite restorations (15). However, in the literature the indication of CAD/CAM resin composite materials is generally limited to permanent single-tooth restorations. Therefore, the question of whether 3D-printed restoration materials, such as resin composites, are suitable alternatives for the indication of use for such multi-unit fixed dental prostheses (FDPs) is an important issue for resin composites with their increasing use in current dentistry (16). Despite the increasing popularity of the 3D printing technique among technicians and dentists, due to its wide variety of materials and related applications as well as ease of use (17), there is currently a lack of evidence regarding the mechanical properties as well as the clinical outcomes of 3D-printed materials designed for both temporary and permanent restorations. Yet, at this moment, no clinical data are available for 3D-printed resin composite 3-unit posterior FDPs. Therefore, the objective of the present study was to evaluate the clinical performance of 3D-printed posterior resin composite FDPs in a short-term follow-up.

## Materials and methods

### Study design

The properties, brands, and manufacturers of the materials used in this study are listed in Table 1. Between 10 October 2020 and 5 August 2022, 49 patients aged 19–60 years (16 men, 33 women; mean age 42.5 years) referred to the Department of Prosthetics Dentistry, Medipol University, Dental School, Istanbul, Turkey, with the indication of at least one 3-unit FDP in the posterior were recruited for this study. A total of 49 patients received 68 3D-printed resin composite 3-unit posterior FDPs in the posterior region of the maxilla or mandible by two operators (HP and OM). Just before participating in this clinical trial, all patients who agreed to participate in the study were provided with a written informed consent form approved by the Republic of Turkey Ministry of Health Turkish Medicines and Medical Devices Agency (Vote number of the Regulatory Ethical Committee No: 68869993-511.06-E.150622; Clinical Trials.Gov identifier: NCT04600297). Each patient was informed about alternative treatment procedures. This study is in compliance with the principles of the Declaration of Helsinki.

**Table 1:** The brand, type, manufacturer, and chemical composition of the main materials used in this study.

Brand	Type (batch)	Manufacturer	Chemical composition
ELS even stronger	3D-printed resin composite	Saremco, Rebstein, Switzerland	Bisphenol A diglycidyl methacrylate ethoxylated, urethane dimethacrylate, trimethylbenzonyldiphenylphosphine oxide; anorganic fillers: dental glass silica
Variolink Esthetic DC	Resin cement	Ivoclar Vivadent, Schaan, Liechtenstein	Bisphenol A diglycidyl methacrylate, urethane dimethacrylate, triethylene glycol dimethacrylate; inorganic fillers: barium glass, ytterbium trifluoride, barium-AL iminium-fluorosilicate glass, spheroid mixed oxide, initiators, stabilizers, pigments. Syntac primer: triethyleneglycol methacrylate, polyethyleneglycol dimethacrylate, maleic acid, ketone; syntac adhesive: polyethyleneglycol dimethacrylate, glutaraldehyde. Heliobond: bis-GMA, triethyleneglycol dimethatcrylate, stabilizers, initiators

### Patient recruitment

The inclusion and exclusion criteria in this clinical observation are listed in Table 2. Two operators independent of the objectives of the study agreed to recruit the patients who required an FDP. After signing the informed consent form, the preoperative status of abutment teeth and their gingival tissues were assessed, and vitality and radiographic assessments were completed. The teeth selected were sound in structure with healthy periodontium.

**Table 2:** The inclusion and exclusion criteria considered in the clinical trial.

Inclusion criteria	Exclusion criteria
Subjects had to be over the age of 18 and agree to keep the scheduled recall appointments for data collection and maintenance and plan to stay in the area for at least 3 years	Subjects suffering from general health impairment or were pregnant during the duration of the study
Subjects had to agree to keep the scheduled recall appointments for data collection and maintenance and plan to stay in the area for at least 3 years	Subjects who are known to be allergic to the ingredients of resin materials
Subjects without obvious untreated caries, dental health problems (regularly checked by a dentist)	Subjects who were restored with a removable partial dental prosthesis (RPDP), unless the RPDP replaced the tooth that was planned to be restored in the study
Subjects with good or moderate oral hygiene (plaque score of less than 30% in anterior region before treatment), No untreated periodontal disease (probing depth and attachment levels within normal limits, no furcation involvement, and no mobility)	Subjects presented with severe wear facets and/or reported parafunctional activities such as clenching or nocturnal bruxism (as the material is being analyzed under normal functional forces). Patients who report parafunctional activities such as clenching or nocturnal bruxism after the delivery of the restoration will also be excluded
Subjects who need for a three-unit posterior FDP with one missing tooth from the second premolar to the second molar	Subjects with considerable periodontal disease without treatment (DPSI3−, 3+, and4)
Only FDPs with opposing natural dentition (either intact or restored with intracoronal or extracoronal fixed restorations), and with a minimum of 20 teeth)	The abutment of FDPs with considerable horizontal and/or vertical mobility of teeth: tooth mobility index score 2 or 3
Only FPDs with end abutments (No cantilever) and sufficient length of the clinical crown of abutments must be over 5 mm	The abutment of FDPs with extensive loss of tooth tissue due to endodontic treatment
Only FPDs with abutment teeth are vital or endodontically treated with a sealed root filling to the apical region, and have to be without apical periodontitis for the past 6 months	

### Clinical procedures

**Figure 1:**
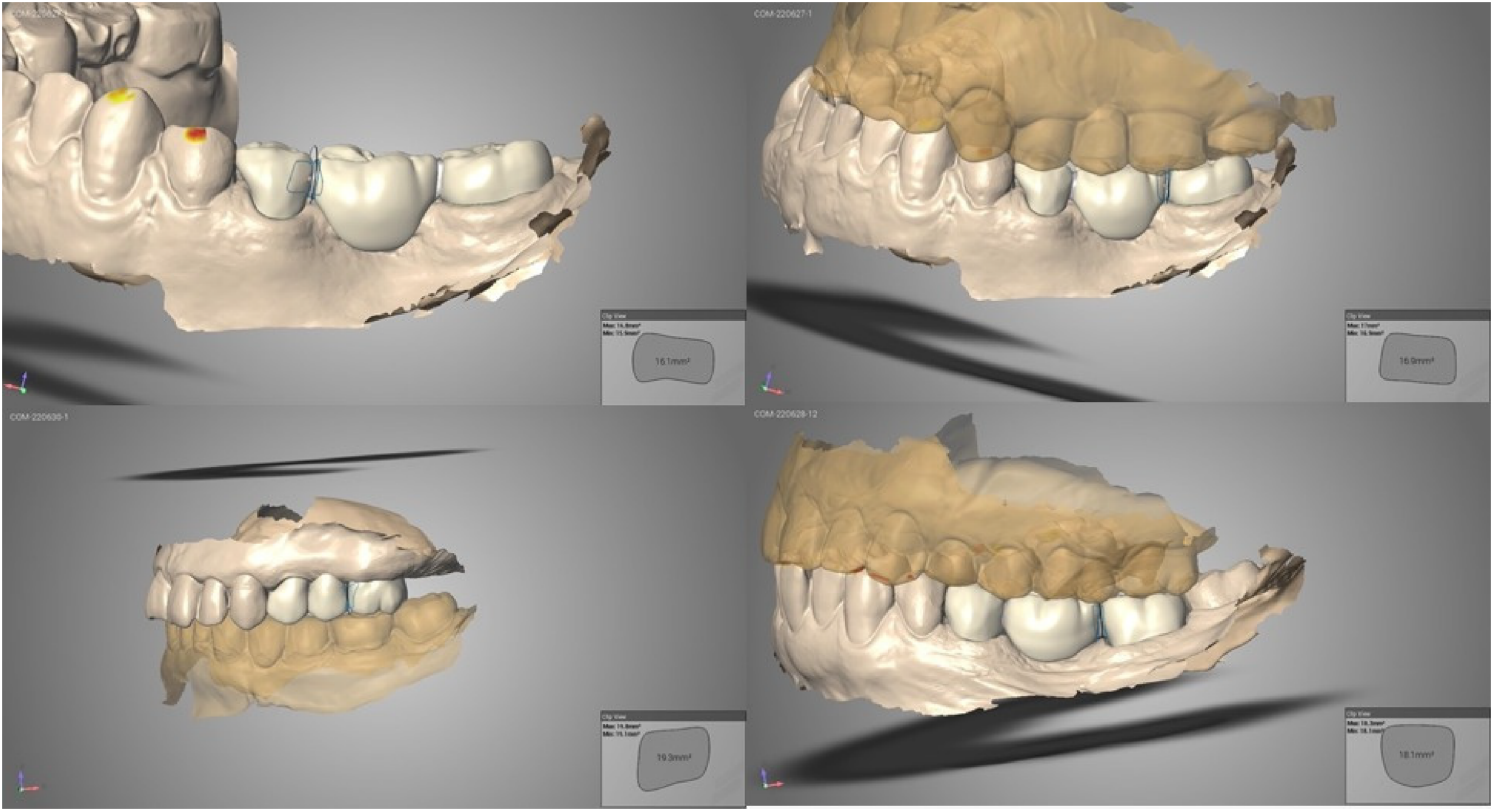
The design of some FDPs demonstrating the shape and the dimensions of the connectors.

Two operators with 15 and 4 years of clinical experience in prosthetic dentistry were appointed to apply all 3D-printed resin composite FDPs. The operators were DDS and PhD candidates in the field of prosthodontics, respectively. Preparation of the teeth was carried out according to the manufacturer's recommendations. The preparation guidelines were applied on the abutment teeth for replacing a missing tooth with the 3D-printed resin composite posterior FDP. The tooth preparation allowed 1.5 mm thickness overall with chamfer finish lines. The minimum length of the clinical crown was 5 mm. The distance difference between the centers of the abutment teeth was applied at a maximum of 20 mm. The minimum occlusal thickness was 1.5 mm and the mesial and distal connector sizes were prepared to be as minimum of 16 mm^2^ (Figure 1). The supragingival or slightly subgingival margins of the preparations respected the biologic width. Digital impressions were acquired using an intraoral scanner (TRIOS 3, 3Shape, Copenhagen, Denmark). The intraoral scanning procedure was performed according to the manual. Resin composite FDPs (ELS Even Stronger, Saremco, Rebstein, Switzerland) were designed using Trios Design Studio and sent to the printer (MAX UV, Asiga, MI, USA) via software (Asiga Composer Software). All resin composite FDPs were checked for marginal fit, inter-proximal contact, and occlusion before cementation. They were then steam-cleaned, dried, washed with isopropanol, and polished according to the manufacturer's recommendations. Photo-polymerization was performed on a polymerization device (Otoflash G171, Nk Optic, Baierbrunn, Germany) according to the manufacturer's recommendations. The resin composite FDPs were cemented using an adhesive resin cement (Variolink Esthetic DC; Ivoclar Vivadent, Schaan, Liechtenstein). Cotton rolls were used to manage salivation and gingival fluid during adhesive placement. A pre-treatment adhesive was applied to the teeth using 37% orthophosphoric acid (Ivoclar Vivadent) and a bonding agent (Adhese Universal). After air abrasion with aluminum oxide (grain size: 50 μm, pressure: 2.5 bar), silane (Monobond Plus, Ivoclar Vivadent) was applied to the intaglio surface. The resin composite FDP was adhesively placed (Variolink Esthetic DC, Ivoclar Vivadent). Excess luting material was removed and the margins of the resin composite FDPs were covered with a protective layer (Liquid Strip, Ivoclar Vivadent) for 8 min. After photo-polymerization with an LED curing light device for 20 s, excess luting material was completely removed.

### Clinical evaluation

Two independent, calibrated clinicians with more than 15–20 years of clinical experience performed the baseline evaluations 15 days after the cementation of the FDPs. The e-calib system (www.e-calib.info) was used for calibration between observers in this research and a minimum inter- and intra-examiner agreement of 80% was accepted (18, 19). Evaluations were performed just 2 weeks after restorative procedures (baseline), and at 6 months and 1 year according to the modified FDI Criteria (18) for anatomical form, secondary caries, marginal adaptation, surface roughness, color match, fracture of material, staining surface, staining margin, approximate anatomical form, retention, gingival health, and patient's view. With regard to periodontal assessment, tooth mobility, plaque accumulation, probing the pocket depth and attachment level, and bleeding on probing (BOP) at the abutment sites (test) and at the contralateral, non-restored teeth (control) were evaluated (Table 3). At the end of the controls, the patients were asked by the observer whether they were satisfied with the esthetic result and the functionality of their FDPs, and the patients’ responses (yes/no) were recorded. The patients were advised to call, in case of any complaints about the FDPs. Failure types were categorized as mechanical and biological ones.

**Table 3:** Clinical criteria according to the modified FDI criteria.

Anatomical form	Approximate anatomical form (contact point)	Secondary caries	Marginal adaptation	Surface luster	Color match	Fracture of material	Gingival health (mesial/facial/distal)	Staining surface	Staining margin	Retention	Patients view
Normal contour	Normal contact point (floss or 25 µm, metal blade can pass)	No secondary or primary caries	Harmonious outline, no gaps, no white or discolored lines	Luster comparable to enamel	Good color match	No fracture	No plaque, No inflammation, No pockets	No surface staining	No marginal staining	Retentive	Entirely satisfied with esthetics and function
Slightly deficient contour	Contact slightly too strong but no disadvantage (floss or 25 µm metal blade can only pass with pressure)	Small and localized	Marginal gap (<150 µm), white lines	Slightly dull, not noticeable from speaking distance	Minor deviations in shade	Small hairline crack	Little plaque no inflammation (gingivitis), no pocket development	Minor surface staining, easily removable by polishing	Minor marginal staining, easily removable by polishing		Satisfied esthetic and function
Visibly deficient contour	Somewhat weak contact, no indication of damage to tooth, gingiva or periodontal structures; 50 µm metal blade can pass	Larger areas, only preventive measures necessary	Gap < 250 µm	Dull surface but acceptable if covered with film of saliva	Distinct deviation but acceptable. Does not affect esthetics	Two or more or larger hairline cracks and/or material chip fracture not affecting the marginal integrity of approximate contact	Difference up to one grade in severity of PBI compared to baseline and compared to control tooth	Moderate surface staining that may also present on other teeth, not esthetically unacceptable	Moderate marginal staining, not esthetically unacceptable		Minor criticism but no adverts clinical effects a-Esthetic shortcomings b-Some lack of chewing comfort c-Unpleasant treatment procedure
Inadequate contour. Repair possible	Too weak and possible damage due to food impaction; 100 µm metal blade can pass	Caries with cavitation. Localized and accessible. Can be repaired	Gap > 250 µm	Rough surface cannot be masked by saliva film, simple polishing is not sufficient. Further intervention necessary	Localized clinical deviation that can be corrected by repair	Material chip fractures which damage marginal quality or approximate contacts	Difference of more than one grade of PBI in comparison to control tooth or increase in pocket depth > 1 mm requiring intervention	Unacceptable surface staining on the restoration and major intervention necessary	Pronounced marginal staining; major intervention necessary for improvement		Desire for improvement (a) Esthetics (b) Function e.g., tongue irritation. reshaping of anatomic form or refurbishing is possible
Inadequate contour Requires replacement	Too weak and/or clear damage due to food impaction and/or pain/gingivitis	Deep caries that is not accessible for repair of restoration	Generalized major gaps or irregularities	Very rough, unacceptable plaque retentive surface	Unacceptable. Replacement necessary	(Partial or complete) loss of restoration or multiple fractures	Severe/acute gingivitis or periodontitis	Severe surface staining and/or subsurface staining, generalized or localized, not acceptable for intervention	Deep marginal staining, not accessible for intervention	Decementation	Completely dissatisfied and/or adverse effects, including pain

### Statistical methods

Data were statistically analyzed using SPSS for Windows version 25.0 statistical software (IBM Corp., Armonk, NY, USA). Qualitative data were recorded in scores and their frequency in percentage. Survival was estimated using Kaplan–Meier analyses.

## Results

**Figure 2:**
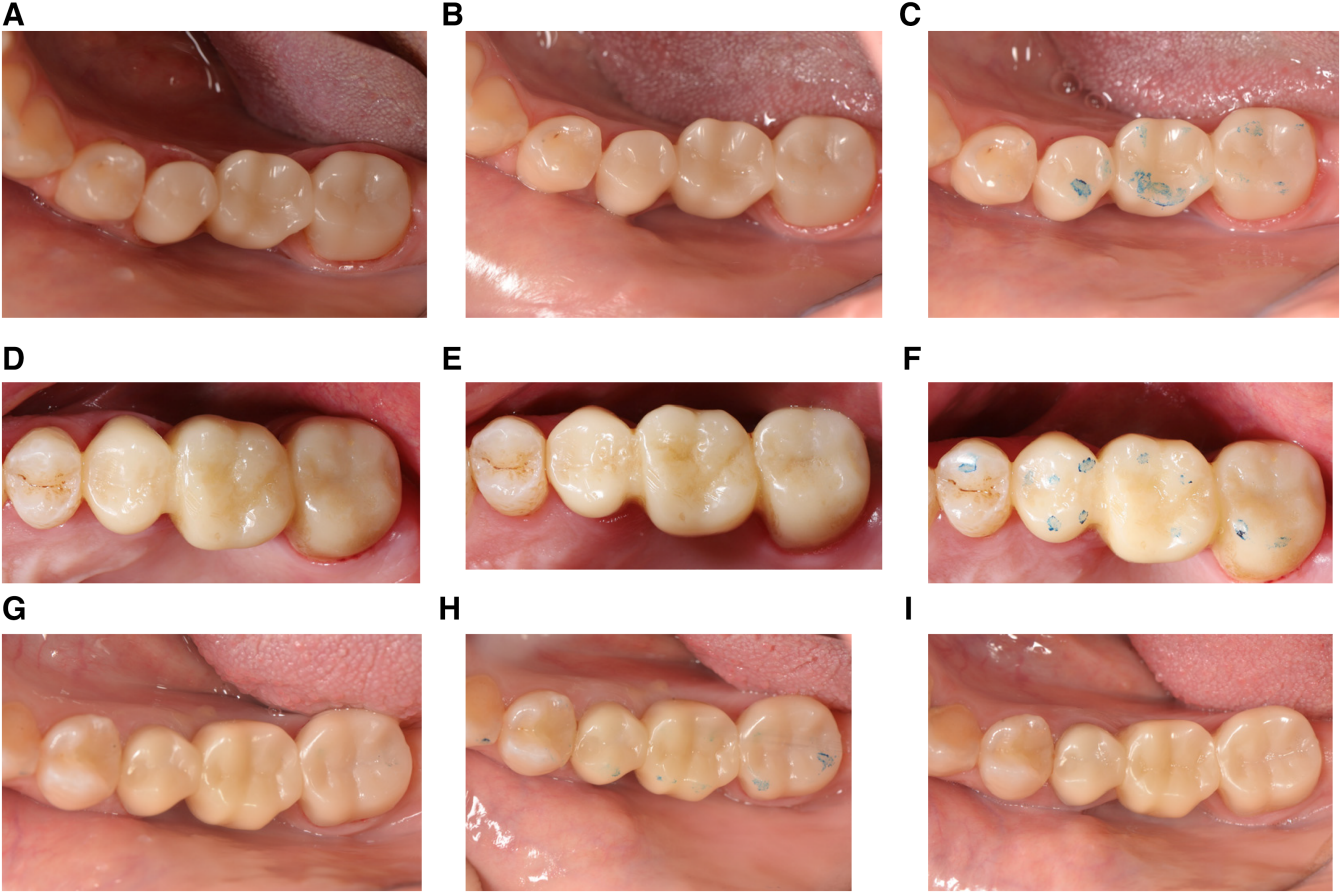
Representative clinical photographs of 3-unit resin composite FDPs (**A**) at baseline, (**B**) 6 months, and (**C**) 1 year from FDP between 35 and 37; (**D**) at baseline, (**E**) 6 months, and (**F**) 1 year from FDP between 15 and 17; (**G**) at baseline, (**H**) 6 months, and (**I**) 1 year from FDP between 45 and 47.

Both operators performed similar numbers of 3D-printed resin composite 3-unit posterior FDPs (HP: *n* = 33; OM: *n* = 35). In total, 68 3D FDPs were cemented in 49 patients. The distribution of the FDPs per location according to the FDI numbering system is presented in Table 4. The mean observation period was 8.63 months (range 1.83–18.7). A total of 59 restorations were evaluated; 45 FDPs completed the 6-month observation period, while the 14 restorations completed the 1-year clinical follow-up period (Figures 2A–I).

**Table 4:** Distribution of the 68 FDPs regarding location of the replaced teeth according to the FDI numbering system.

Replaced tooth	16	15	25	26
Number of FDPs	5	7	4	2
Replaced tooth	46	45	35	36
Number of FDPs	19	2	4	25

**Figure 3:**
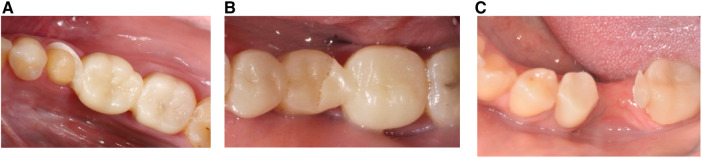
Representative clinical photographs from failures of 3-unit resin composite fixed dental prostheses. (**A**) Cohesive failure in 35–37 after 2 months; (**B**) cohesive failure in 45−47 after 6 months; and (**C**) connector fracture at both abutment sides 45−47 after 6 months.

**Figure 4:**
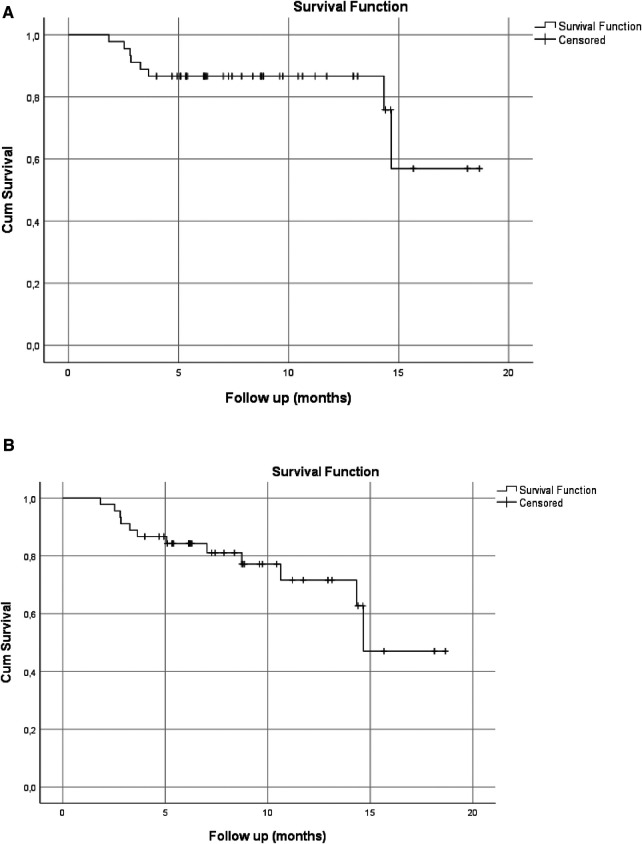
(**A**) Event-free survival rates of 3-unit resin composite restorations FDPs (*N* = 68) with 86.7% (95% CI: 76.7–96.7) based on mechanical complications only. (**B**) Event-free survival rates of 3-unit resin composite restorations FDPs (*N* = 68) with 71.6% (95% CI: 55.1–88.1) based on combined mechanical and biological complications.

In the evaluations, a total of 14 FDPs were found to be clinically unsuccessful. When the failures were classified as biological and mechanical, five biological failures and nine mechanical failures were encountered. The observed biological failures occurred within the first 6 months with the need for root canal treatment and gingival tissue damage. Three patients complained of pain and were diagnosed with irreversible pulpits at 2, 4, and 9 months after treatment. Two of the resin composite FDPs presented gingival problems that could not be accepted as clinically successful and needed replacement. In the intraoral evaluation of the patients, bleeding was observed in the gingiva along with gingival recession buccally. As the restorations were cemented using an adhesive resin cement, they were cohesively fractured during removal. As a result, a new 3D-printed resin composite FDP was luted in these two patients after the gingival tissue was healed (Table 5). Mechanical failures were generally observed as connector fractures. Eight FDPs showed cohesive fractures of which seven were in one connector, either in the mesial or distal site only and one in the pontic itself (Figures 3A–C). Six of these fractures were observed during the 6-month follow-up period and two were detected during the 1-year follow-up period. One FDP showed loss of retention within the 6-month control period. When the failures were considered in terms of replacement teeth, 12 of the 14 failures in total (9 mechanical, 5 biological) were observed in the replacement of a molar and 2 were premolar. Based on mechanical failures, the survival rate was 86.7% [95% confidence interval (CI): 76.7–96.7] and when biological complications were included, the survival rate was 71.6% (95% CI: 55.1–88.1) (Kaplan–Meier) (Figures 4A,B). Considering the anatomic forms of the resin composite FDPs, it was determined that all restorations were in normal anatomical contour during the follow-up period and all restorations had normal contact points. At the 6-month recalls, one resin composite FDP showed a dull surface. Moderate surface staining was assessed in three FDPs after 6 months and unacceptable surface staining was observed in two of the resin composite FDPs after 1 year due to the patients’ smoking habits. However, these stainings were also present in other teeth and the patients were satisfied with the esthetic and function of their resin composite FDPs. Thus, the discolorations did not require intervention. After 1 year, two FDPs showed surface luster loss (score 4), two-color mismatches (score 4), and two-surface staining (score 4) according to FDI criteria (Table 6).

**Table 5:** Life table of mechanical and biological failure types observed in the FDPs.

No	Failure type	Failure time	Failure reason	Failure case	Replacement tooth
1	Biological Failure	6 months	Gingival failure	45–47 FDP	Molar
2			Gingival failure	35–37 FDP	
3			Endodontic treatment	45–47 FDP	
4			Endodontic treatment	35–37 FDP	
5			Endodontic treatment	34–36 FDP	Premolar
6	Mechanical failure	6 months	Fracture (connector)	45–47 FDP	Molar
7			Fracture (connector)	45–47 FDP	
8			Fracture (connector)	35–37 FDP	
9			Fracture (connector)	45–47 FDP	
10			Fracture (connector)	35–37 FDP	
11			Fracture (Pontic)	45–47 FDP	
12			Retention failure	35–37 FDP	
13		1 year	Fracture (connector)	45–47 FDP	
14			Fracture (connector)	34–36 FDP	Premolar

**Table 6:** Distribution and percentage of scores for resin composite FDPs (*N* = 59) according to the FDI criteria that could be evaluated at 6 months and 1 year.

Criteria	Distribution and percentage (%) of scores for resin composite FDPs (*n* = 59)				
	1	2	3	4	5
Anatomical form	42 (71%)	2 (3%)	2 (3%)	0 (0%)	0 (0%)
Approximal anatomical form (contact point)	45 (76%)	0 (0%)	0 (0%)	0 (0%)	0 (0%)
Secondary caries	45 (76%)	0 (0%)	0 (0%)	0 (0%)	0 (0%)
Marginal adaptation	39 (66%)	2 (3%)	4 (7%)	0 (0%)	0 (0%)
Surface luster	37 (63%)	5 (9%)	1 (2%)	2 (4%)	0 (0%)
Color match	40 (68%)	2 (3%)	0 (0%)	3 (5%)	0 (0%)
Fracture of material	45 (76%)	0 (0%)	0 (0%)	0 (0%)	0 (0%)
Gingival health	25 (42%)	12 (20%)	8 (14%)	0 (0%)	2 (3%)
Staining surface	36 (42%)	3 (7%)	3 (7%)	3 (7%)	0 (0%)
Staining margin	36 (42%)	4 (7%)	4 (7%)	1 (2%)	0 (0%)
Retention	45 (76%)	0 (0%)	0 (0%)	0 (0%)	1 (2%)
Patient’s view	45 (76%)	0 (0%)	0 (0%)	0 (0%)	3 (7%)

## Discussion

After the introduction of digital technology to dentistry, the idea that these innovations could enable more conservative and long-term treatment alternatives has led to new ideas in terms of prosthetic approaches. Therefore, there is the question of whether resin composite 3D-printed restoration materials might be appropriate alternatives for the indication of 3-unit FDPs. With this question, the objective of this clinical evaluation was to assess the clinical outcome of 3D-printed posterior resin composite FDPs.

There are various studies in the literature evaluating the success of different prosthetic restorations under *in vitro* conditions (6, 20); however, the reports on the clinical performance of FDP materials are scarce. At the same time, no clinical study has yet been conducted to evaluate the success of 3D-printed resin composite posterior FDPs. In a laboratory study, it was realized that fractures for FDPs generally occurred within the connector starting at the gingival interdental embrasure (20). *In vitro* and finite element studies have revealed that cracks and fractures for FDPs originate from the gingival surface of the connector, as under the tensile loading weak point was toward the pontic (21). In the current study, eight of the resin composite FDPs with fractures were observed in one connector, either in the mesial or distal site only.

Zimmermann et al. (20) tested the fracture strength of ceramic and composite 3-unit FDPs fabricated with subtractive and additive CAD/CAM technologies. They found the lowest fracture load for ELS Even Stronger fabricated FDPs when compared with other restorations, such as zirconia, lithium disilicate, CAD/CAM composite, and polymethyl methacrylate (20). Clinical studies evaluating the CAD/CAM techniques revealed that the connector area dimensions of FDPs are the most influential fracture failures (22, 23). The failure rate is generally higher with 3-unit all-ceramic FPDs around the sharp connector area, particularly where connector sizes are reduced for biological and esthetic reasons (24). Recommended minimum connector cross-sectional area specified in the literature is 12–16 mm^2^ (25). In the present study, a total of six cohesive fractures were observed in FDPs during the 6-month follow-up. Most of these fractures were observed at the connector cross-sectional area. This finding was in line with the study by Wimmer et al. (26), which concluded that CAD/CAM resin FDPs showed a prominent increase in fracture load with the increase of the connector cross-sectional area.

Since 3D-printed resin composites are considered stronger than conventional resin composites, they are often preferred in FDPs. It is reported in the literature that resin composite restorations are known to accumulate more plaque than enamel and other type of restorations and this can lead to caries formation and gingival problems (27). In the present study, two resin composite FDPs were replaced due to gingival problems. During the intraoral examination of the patients, bleeding was observed in the gingiva along with gingival recession observed in the buccal areas. This clinical observation is mostly related to the deep-margin preparation of the teeth, especially for metal-ceramic replacements. The surface roughness of restorations may also affect plaque biofilm adhesion. In this study, one standard polishing procedure was applied to resin composite FDPs. Therefore, different polishing procedures, through which the surface roughness can be achieved at more optimum levels, may decrease dental plaque accumulation and thereby better soft tissue conditions.

The superior mechanical properties of resin composites depend on the conversion of a large percentage of the monomers to polymers during polymerization and, thereby obtaining an adequate degree of conversion (28). 3D printers especially the ones using DLP technology, use a different process that may affect the polymerization degree of the printed materials. As the layers of this printing technology are formed by photo-polymerization via an LED device, the difference in layer thickness influences the light penetration into the resin material and changes the degree of the conversion. In the present study, layers of 50-μm thickness were polymerized with the automatically selected parameters depending on the chosen resin during the printing process. Since the emission of light on the incrementally added layers of monomer can affect the quality of the printed part, a lower thickness layering may improve the mechanical and biological properties with a lower amount of plaque accumulation of the tested material. In a laboratory evaluation, three different printing layer thicknesses (25, 50, and 100 μm) were evaluated using different polymerization protocols with the results being more favorable for the 25 μm layer thickness with a higher degree of conversion after being heat-polymerized for 15 min (29). Yet, 3D printers today are programmed in such a way that the transition between the layers is smoothened and the surface properties are significantly affected by the subsequent cleaning and post-polymerization processes. Thus, this aspect needs further investigation.

The organic and inorganic composition of the materials can be accepted as a different factor affecting the surface roughness, wettability, and microbial adhesion to restorative materials. The tested 3D-printed resin composite in the present study includes bis-EMA as a hydrophobic monomer and bis-EMA is mentioned in the literature with lower water absorption and a relatively higher degree of conversion (30). Bis-EMA is a dimethacrylate analog to bis-GMA in which the hydroxyl functionalities in the molecular structure are replaced by ethoxy groups (–O–CH_2_CH_3_) and is pronounced as a class of monomers with several ethoxylations in the chain. The higher the number of ethoxylations, the larger the molecule and the greater the hydrophilicity (31). The monomer content of the 3D resin composite FDPs may result in less surface degradation in the long term due to the hydrophobic nature of the restoration, and the higher conversion degree of the resin composite that may cause less plaque accumulation.

One of the resin composite FDPs, which was renewed due to the gingival problem, was carried out to replace an old prosthetic treatment due to periodontal problems. Although initially no infection was present, there was the possibility that the gingival failure of the resin composite FDP was caused by the oral hygiene of the patient rather than plaque accumulation in the restorative material. The purpose of applying full-coverage restorations is to preserve the prepared tooth in its original state as much as possible, following the principles for tooth preparation, impression taking, crown making, and cementation. The position of the finishing line in relation to the gingival margin has a great effect on periodontal behavior around teeth supporting prosthetic restorations. It should be kept in mind that subgingival margins may result in inflammatory periodontal reactions (32). Thus, the gingival problems of the 3D resin composite FDPs could be related to the deep preparation protocol performed in the present study. The role of the abutment might be especially crucial for the evaluation of the success of FDPs regardless of the material type. In the present study, one resin composite FDP showed adhesive retention failure. The failure was attributed to a larger taper angle due to previous tooth preparations. The restoration was cleaned with air abrasion and recemented according to the manufacturer's instructions. In cases where there is no need for prosthetic treatment renewal, the required thickness of the connectors, which are areas where fracture-type failures are generally observed, are given by the manufacturers for the durability of FDPs. This is critical in posterior FDPs, where connector height is usually limited by short clinical molar crowns. However, the connector area thickness given by the manufacturers in particular may direct the dentist to increase the area by making deeper preparations in posterior FDPs (33). Providing the required 16 mm^2^ area in our study may be considered a limitation for short posterior teeth.

In the present study, endodontic treatment was applied to three abutment teeth as a result of excessive postoperative sensitivity during the first 6 months of clinical observation. Restorations were removed and endodontic treatment was performed. As the restorations were cohesively fractured during removal, new 3-unit FDPs were manufactured and recemented. Crown restorations and/or FDPs are usually indicated for the restoration of grossly damaged teeth associated with the exposure of large amounts of tooth tissue. The adhesive cementation procedure with substantial exposure of dentin structure is regarded among the most challenging tasks of FDPs (34). The cementation process was considered difficult for choosing the appropriate resin cement to be used as many adhesive systems have been introduced to achieve good adhesion between resin cement and tooth tissues. Resin cements are generally classified according to tooth substrate pre-treatment into etch-and-rinse, self-etch, and self-adhesive (35). The preferred method in cementation in this study was the etch-and-rinse technique, and the excessive postoperative sensitivity observed in the abutment teeth in the early period could be associated with the application of acid to the deep dentin tissues. Acid application leads to the removal of the smear layer, exposure of dentinal tubules, dentinal fluid movement, and penetration of uncured monomers to the pulp tissue. However, the etching procedures are not performed in the case of self-etch and self-adhesive resin cement where the remaining smear layer acts as a protective layer and achieves a superficial chemical interaction between the adhesive material and tooth tissue (36). Different clinical observations performed with the application of self-etch adhesive systems in the cementation procedures may result in better postoperative sensitivity data.

The proximal contact points of FDPs are known to play an important role in protecting and stabilizing the dental arch. In line with this information, it should be borne in mind that poor contact points can, in some cases, cause food embedding, dental caries, periodontal disease, failure of occlusion, and undesirable shifting of teeth. On the other hand, in the presence of very tight contacts, it should be noted that the periodontal tissues are damaged, and these contacts cause improper tooth movement or interfere with the physiological placement of the teeth. Clinical evaluations of proximal contact points were performed with dental floss at the time of cementation and after recalls as in the previous study (37). Dental floss was passed with little or as much resistance as in natural dentition on the other side in the observations and the contact points were considered as acceptable. *In vitro* investigations showed mechanical and chemical degradations for CAD/CAM composite materials due to higher water uptake and thermal expansion compared with ceramic ones (38). Accordingly, two FDPs required polishing due to the surface roughness and surface staining after 1 year in clinical service. The discolorations were related to the patients’ smoking habit but did not interfere with patient satisfaction and thereby replacement of the FDP.

In this study, the success of 3D-printed resin composite, whose indication is limited to single crowns, is evaluated in 3-unit posterior FDPs. Since biological failures depend on either the status of the abutment teeth or on patient-related factors, the failures may not reflect the real failure due to the material itself. A long-term clinical follow-up of the present cohort will indicate whether the tested resin composite material may serve as long-term provisional or even as permanent restorative material for 3-unit FDPs. The cost of the tested material is significantly less than that of its counterparts. Yet, possible failure types need to be communicated with the patient. To the best of our knowledge, to date, no clinical trials are available with the tested material. A longer follow-up will also focus on the analysis of the location and the tooth type on the survival.

## Conclusions

The findings of this study indicated that 3D-printed resin composite FDPs with the tested resin composite material were acceptable after 1 year of clinical follow-up, providing that the experienced failure types were mainly associated with fractures in the connector region, which requires a revision of the design parameters. The 3D resin composite FDPs evaluated in this study exceeded the expectations from a temporary material and can thus be considered to be long-term provisional restorations. The FDPs are being followed up for long-term survival to find out whether they may serve as permanent FDP alternatives.

## Data Availability

The original contributions presented in the study are included in the article/Supplementary Material, further inquiries can be directed to the corresponding author.
